# Mechanical and Corrosion Behavior of Zr-Doped High-Entropy Alloy from CoCrFeMoNi System

**DOI:** 10.3390/ma16051832

**Published:** 2023-02-23

**Authors:** Santiago Brito-Garcia, Julia Mirza-Rosca, Victor Geanta, Ionelia Voiculescu

**Affiliations:** 1 Mechanical Engineering Department, Las Palmas de Gran Canaria University, 35017 Tafira, Spain; 2 Faculty of Industrial Engineering and Robotics, Politehnica University of Bucharest, 313 Splaiul Independentei, 060042 Bucharest, Romania

**Keywords:** HEA, CoCrFeMoNiZr, microstructure, corrosion, EIS, Young modulus

## Abstract

The aim of the paper is to study the Zr addition effect on the mechanical properties and corrosion behavior of a high-entropy alloy from the CoCrFeMoNi system. This alloy was designed to be used for components in the geothermal industry that are exposed to high temperature and corrosion. Two alloys, one Zr-free (named Sample 1) and another one doped with 0.71 wt.% Zr (named Sample 2), were obtained in a vacuum arc remelting equipment from high-purity granular raw materials. Microstructural characterization and quantitative analysis by SEM and EDS were performed. The Young modulus values for the experimental alloys were calculated on the basis of a three-point bending test. Corrosion behavior was estimated by linear polarization test and by electrochemical impedance spectroscopy. The addition of Zr resulted in a decrease in the value of the Young modulus but also in a decrease in corrosion resistance. The beneficial effect of Zr on the microstructure was the grain refinement, and this ensured a good deoxidation of the alloy.

## 1. Introduction

Since 2004, when Yeh et al. [1] and Cantor et al. [2] published the results of multicomponent equiatomic alloys, eventually called high-entropy alloys (HEAs), combinations of many components have been developed due to their improved properties compared to classic alloys (of a main element with more than 50 at.%). Yeh et al. defined these alloys as containing a minimum of five components that account for between 5% and 35% by atomic weight.

Cantor was the first to create an equiatomic alloy from the CoCrFeMnNi system, and it was observed that the mechanical characteristics of high-entropy alloys rely on chemical composition and microstructure [2,3]. If certain thermomechanical treatments are applied to this alloy, its mechanical properties can be considerably improved. For example, after treatment, the yield strength reaches up to 1834 MPa and the ultimate tensile strength rises to 2069 MPa, with uniform elongation of 1.4% and total elongation of 7.4% at room temperature. This alloy has high fracture resistance and low-temperature ductility, although there have been only few investigations on how it react to corrosion. In some chemical conditions, such as those containing chlorides, the corrosion resistance of this alloy is not noticeably higher even if the amount of Cr and Ni is higher than that of stainless steels (such as AISI 304) [4,5,6].

The elements selected for the CoCrFeMnNi alloy belong to the 3d and 4d transition metal groups. In Fe-base alloys, Cr is a passivating element [7,8]; together with Ni, it provides good corrosion behavior in line with the main elements in stainless steels. By increasing the Cr content from 4.76 at.% to 33.36 at.% in AlCrCoFeNi alloy, the hardness increases from 390 to 551 HV0.1, and a good combination of strength and ductility is obtained [9,10,11]. Co also improves the high-temperature resistance and tensile properties [12,13,14,15]. The high ultimate tensile strength (UTS) of multielement CoCrNi alloy is about 740 MPa; however, after grain size refinement, it can reach 1000 MPa. Furthermore, the elongation at fracture of this alloy is about 50%. The yield strength can be increased to about 1 GPa in the case of the CoCrNi–3W alloy, which is almost double compared to the CoCrNi alloy (400–500 MPa) [16]. The energy threshold for the mobility of Mn atoms is usually lower than that of Co and Ni atoms, which allows it to diffuse more easily [17] but the corrosion resistance decreases [18]. The alloying of CoCrFeNi with Nb and Mo allows to obtain of yield strength of 426 MPa and a fracture strength of 714 MPa, with elongation of 17.4% [19].

We decided to modify the alloy recipe proposed by Cantor using another element instead of Mn, such as Mo, to increase the yield strength, ultimate strength, and hardness. Some researchers used metalloids, such as Si and Ge, to simultaneously increase the yield strength, ultimate strength, and ductility, due to the supplementary covalent bonding and reinforcement effect of solid solution [20,21,22].

The replacement of Mn with tungsten, reported for the CoCrFeNiWx alloy, caused an increase in hardness proportional to the W concentration [23].

It was found that, by reducing the Mn concentration (to 5 at.% Mn), the CoCrFeMnNi alloy acquires higher yield strength values after annealing at 1117 K (from 387 to 477 MPa) due to the grain refinement effect (from 4.4 to 2.61 µm) [10]. Mn also shows a rapid evaporation effect during melting processes; hence, in our study, this element was replaced by Mo, which could improve the resistance to high temperatures [24]. Co–Cr–Mo alloy is widely used for manufacturing of vanes and gas turbines due to its excellent fatigue behavior and wear resistance [25,26,27,28]. In such alloys, molybdenum has a beneficial effect on passivation capacity, contributing to the formation of a protective surface layer that allows pitting corrosion to be limited [29,30]. Studies were conducted to estimate the effects on the microstructure and properties of alloys with different Mo contents [31,32,33]. Such alloys have been designed for the manufacture of furnace casings and chemical tanks used in high-temperature and corrosion conditions [34], for components used in the geothermal industry [35,36], or to obtain high-performance coatings [37].

The addition of Zr to multielement alloys allows the improvement of some mechanical properties [38]; thus, in TiMoNbZrx alloy, Zr determined the improvement of wear resistance, due to the formation of an oxide film that acts as a lubricant [39]. In terms of corrosion resistance, grain refinement plays a great role, and a small addition of Zr can contribute to grain refinement [40]. Thus, our study focuses on the use of the Mo and Zr in an HEA for the manufacture of mechanical parts used in the geothermal industry at high temperature and in corrosive media.

The impact of Zr addition on the CoCrFeNiMo high-entropy alloy’s microstructure, elastic modulus, and corrosion characteristics is examined in this work. In order to analyze the microstructure of the samples with and without Zr, optical microscopy, scanning electron microscopy (SEM), and energy-dispersive X-ray spectroscopy (EDS) are employed, microhardness tests are performed, a three-point bending test is used to evaluate the modulus of elasticity and linear polarization (LP), and electrochemical impedance spectroscopy (EIS) is applied to examine electrochemical responses, corrosion kinetics, and interface parameters.

## 2. Materials and Methods

### 2.1. Material Preparation

On the basis of the CoCrFeNiMn combination, Mn was replaced by Mo, and a high-entropy alloy was created, generating Sample 1. To improve the mechanical properties, this alloy was then doped with Zr in a proportion of 0.71% by weight, creating Sample 2. The elements utilized to create these alloys were 99.00% pure, and the resulting alloys contained the weight percentages and atomic percentages given in Table 1.

The ingots of each alloy were prepared at LAMET laboratory, Romania, with VAR (vacuum arc melting) model MRF ABJ 900 VAR (Allenstown, Merrimack, NH 03275, USA), under a protective argon atmosphere, and six remelting operations were performed for complete homogenization.

The samples were prepared from ingots after cutting and embedding in epoxy resin. The specimens were ground using emery discs on a Tegrapol-11 Struers (Copenhague, Denmark) polishing machine, and then each sample was polished applying a 0.1 µm alumina suspension on a polishing cloth until it yielded a mirror-like surface, without scratches. Following an ethanol wash, the samples were rinsed with distilled water. The electrical contact between the sample and the potentiostat connection clamp was established using a copper wire.

Using ImageJ, version 1.53 k (public domain), each sample’s surface area S was calculated; for this, 10 measurements each were taken. For each alloy, the equivalent weight *E_w_* (in g/eq) and density ρ (in g/cm^3^) values were also computed (see Table 2).

In this case, the equivalent weight of each sample was calculated using the atomic weight of each element (*M_i_*), its valence number (*n_i_*), and *C_i_* (its weight percentage), as shown in the following equation:

(1) $$E_{w}=\overset{k}{\underset{i=1}{\sum }}\frac{M_{i}}{n_{i}}\cdot C_{i}.$$

The alloy density was calculated as follows:

(2) $$\rho =\overset{k}{\underset{i=1}{\sum }}\rho_{i}\cdot C_{i},$$

where ρ*_i_* (g/cm^3^) is the density of each element, and *C_i_* is its weight percentage.

### 2.2. Microstructural Characterization

The microstructure of the alloys was analyzed using an environmental scanning electron microscope (ESEM) model Fei XL30 ESEM (MTM, Leuven, Belgium) equipped with a LaB6 cathode coupled to an energy-dispersive X-ray electron probe analyzer (EDAX Sapphire) used to determine the chemical composition of the samples. Prior to microstructural characterization, the samples were prepared according to ASTM E3-11 (2017) standard for metallography [41]. Phase analysis was characterized using an X-ray diffractometer (XRD) with an Empyrean diffractometer (Malvern-Panalytical). For the analysis CuKα radiation (1.5406 Å) in the range of 2θ = 30°–100° with a step size of 0.04° at a power of 45 kV was used.

### 2.3. Modulus of Elasticity and Microhardness

A three-point bending test was performed to obtain the modulus of elasticity of the material. For this purpose, the samples were cut with an IsoMet^®^ 4000 Buehler precision linear saw (BUEHLER, Lake Bluff, IL, USA), which is capable of achieving minimal distortion of the material. Ten specimens were selected from each alloy to obtain a weighted average of the results; the specimens had the shape of rectangular section filaments of variable dimensions due to the irregular shape of the ingot obtained in the casting of the alloy. The support spacing (L) in the test for Sample 1 and Sample 2 was 12.70 mm and 8.95 mm, respectively.

Once the samples were obtained, a three-point bending test was performed using the Electroforce 3100 equipment (BOSE Corporation, Eden Prairie, Minnesota, USA), until material breakage was reached or until the maximum equipment load of 22 N was applied, all in compliance with ISO 7438:2020 [42].

Microhardness tests were performed at 24 °C and 48% humidity using a Shimadzu HMV 2T microhardness equipment (Tokyo, Japan). According to ISO 14577-1:2015 [43], 10 indentations were made for each sample, and a load of 1.96 N was applied.

### 2.4. Electrochemical Measurements

Sample 1 and Sample 2 were subjected to corrosion tests in a 3.5% NaCl solution using Biologic SP-150 potentiostat (Seyssinet-Pariset, France). For the execution of the techniques and the establishment of the process parameters, the EC-Lab^®^ v-9.55 program was employed, which also allows the plotting of the data obtained, as well as the calculation of the polarization resistance (Rp), the Tafel coefficients, and the remaining electrochemical parameters.

The tests were carried out in a standard electrochemical cell with three electrodes, consisting of the sample to be tested as a working electrode, a saturated calomel electrode (SCE) as the reference electrode, and a platinum electrode as the counter electrode. Previously, the open-circuit potential was recorded by immersing the samples for 24 h in the salt solution. These tests were performed three times to ensure reasonably reproducible quality.

The linear polarization test establishes the linear relationship between the applied polarization and the current response in the vicinity of the corrosion potential (E_corr_). In this case, the potential range of 25 mV ± 1 versus E_corr_ and a sweep rate of 0.166 mV/s were used, and the polarization resistance was calculated. For potentiodynamic polarization curves, a scan of potential from −1.2 V vs. SCE to +1.2 V vs. SCE was performed.

Additionally, the EIS test was performed using single sine wave measurements at frequencies in the range of 10^−^^1^ to 10^5^ Hz for the two alloys. From this test, it was possible to relate the chemical and physical properties of the prepared alloys to the electrochemical process taking place by analyzing the spectra obtained. Therefore, the ZSimpWin 3.22 program (AMETEC, Princeton, NJ, USA), which allows the interpretation of the EIS data, was used to obtain and analyze the spectra. In addition, from the obtained data, an equivalent circuit was fitted in order to interpret the behavior of the electrolyte/sample interface and the state of the surface layer.

## 3. Results and Discussion

### 3.1. Microstructural Characterization

**Figure 1:**
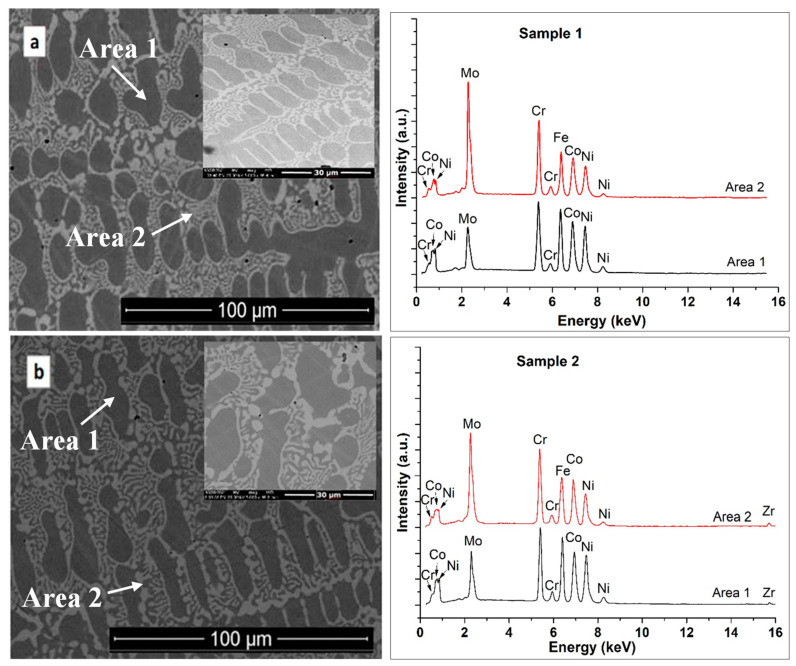
SEM micrographs and corresponding EDS spectra for sample 1 (**a**) and sample 2 (**b**).

Representative SEM images of the two HEAs are presented in Figure 1a,b, both of which showed a compact microstructure, without cracks. The microstructure of both alloys was dendritic, with a grain refinement tendency in the case of Sample 2. A semi-quantitative analysis was performed in order to reveal the chemical composition of the investigated samples on the micro-areas labeled Area 1 and Area 2. Table 3 presents the quantified values and estimated uncertainties of the elements found in the alloys’ composition. From the EDS analysis, it can be observed that the dendritic zone, labeled Area 1 in both samples, was rich in Ni, Fe, and Co. Area 2, corresponding to the interdendritic space, was made up of alloy rich in Cr and Mo, with the concentration of elements such as Co, Fe, and Ni being slightly lower. Low concentrations of Zr were identified in Sample 2, with slightly higher concentrations in the interdendritic areas. A comparative analysis of the microstructure of the two alloys highlights that the volume fraction of the interdendritic zones decreased when Zr was added to the CoCrFeMoNi alloy. In this type of alloy, the σ phase frequently appeared alongside the FCC structure. The addition of Mo to the CoCrFeNi system caused the formation of eutectic containing intermetallic phases (σ and µ) in the FCC phase. The eutectic microstructure influences the mechanical properties, determining the increase in hardness and yield strength [44]. The key elements favoring the appearance of the σ phase are Mo and Cr, whose concentrations are higher in the interdendritic zone [44]. In the high-entropy alloy CoCrFeNiMo, about 14% Cr- and Mo-rich σ phase was identified [19]. In our alloys, the addition of 0.48 at.% Zr caused the Mo concentration to decrease from 20 at.% to 17 at.% in the interdendritic zones, while the Cr concentration remained almost unchanged, at approximately 25 at.%. In this way, the tendency to form the sigma phase was diminished.

**Figure 2:**
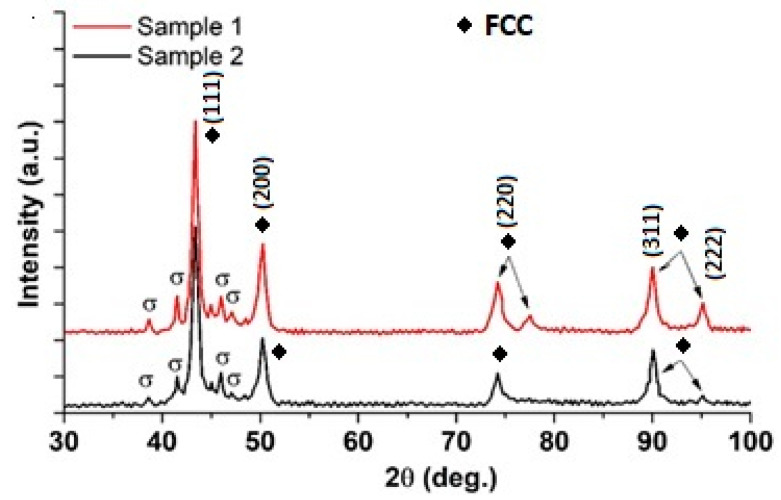
XRD patterns of the investigated alloys.

Table 3 shows the chemical composition determined using the EDS method, where a higher concentration of Mo and Zr in the interdendritic areas can be noted. The X-ray diffraction patterns for both investigated samples are shown in Figure 2. The alloys presented an FCC solid solution with some weak peaks, in addition to the (111) high-intensity peak. Thus, the lower-intensity peaks were attributed to the secondary σ phase, corresponding to the rich Cr- and Mo-rich areas determined in the EDS investigations.

### 3.2. Modulus of Elasticity and Microhardness

**Figure 3:**
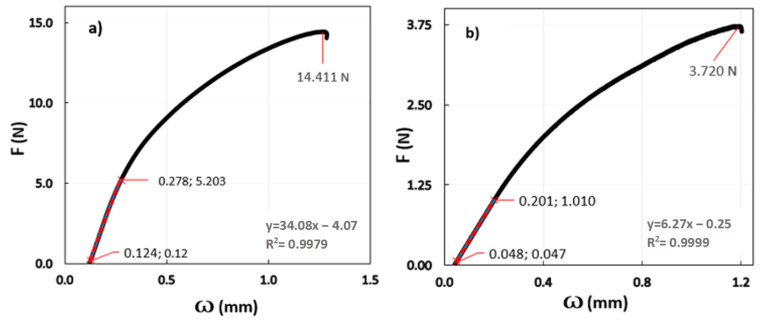
Three-point flexural test diagrams: (**a**) Sample 1 and (**b**) Sample 2.

The load–displacement diagrams were obtained for the two samples using the three-point bending test. Figure 3 shows one of the graphs for each specimen (10 samples of each alloy were tested) and the straight-line results within the elastic limit of the alloy (i.e., when the sample recovers its initial shape after deformation). The gradient of this line is

(3) $$\frac{dF}{dw}=\frac{48EI}{L^{3}},$$

where *E* is the Young’s modulus of the sample of length *L*, which rests on two roller supports and is subject to a concentrated load *F* at its center having a central deflection *w*. *I* is the second moment of area, defined by

(4) $$I=\frac{a^{3}b}{12},$$

where *a* is the sample’s depth, and *b* is the sample’s width. Thus, the average value of the modulus of elasticity of each of the proposed alloys was calculated. The obtained results highlight that the alloy without Zr had a higher modulus of elasticity than that to which Zr was added, as presented in Table 4.

It was found that the value obtained for Sample 1 was 20.5% higher than the value reached by the Sample 2; the addition of Zr produced a decrease in the modulus of elasticity and, therefore, in its stiffness.

**Figure 4:**
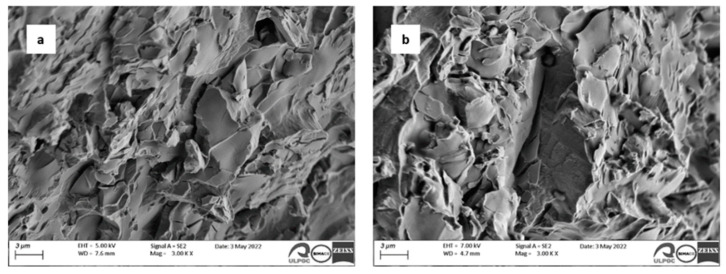
SEM images of the fracture after three-point bending test: (**a**) Sample 1 and (**b**) Sample 2.

The microhardness values HV0.2 and the standard deviation for the 10 measurements of each sample are presented in Table 4. It can be observed that the addition of Zr improved the average microhardness of HEAs. The SEM images of the fracture surfaces of the two alloys are presented in the Figure 4; it can be observed that that a brittle fracture occurred in both samples. A cleavage-like fracture plane can also be observed.

### 3.3. Electrochemical Measurements

The samples were immersed in 3.5% NaCl solution, and the open-circuit potential (OCP) was recorded as a function of time up to 24 h. The OCP values changed continuously, fluctuating more rapidly during the first hours of immersion and reaching relatively stationary values only after 24 h. Was observed that the OCP values after 24 h of immersion were negative for both Sample 1 (−342 ± 63 mV vs. SCE) and Sample 2 (−458 ± 43 mV vs. SCE). This negative shift was related to the alteration of the film from the sample surface. The steady-state potentials corresponding to the corrosion potential (E_corr_) were obtained. Then, the linear polarization test in a potential range of 25 mV ± 1 versus E_corr_ was performed, and the polarization resistance R_p_ was obtained. Tafel measurements were started from the cathodic to anodic direction in the range of −1.2 V to +1.2 V vs. SCE in order to obtain the Tafel slopes.

**Figure 5:**
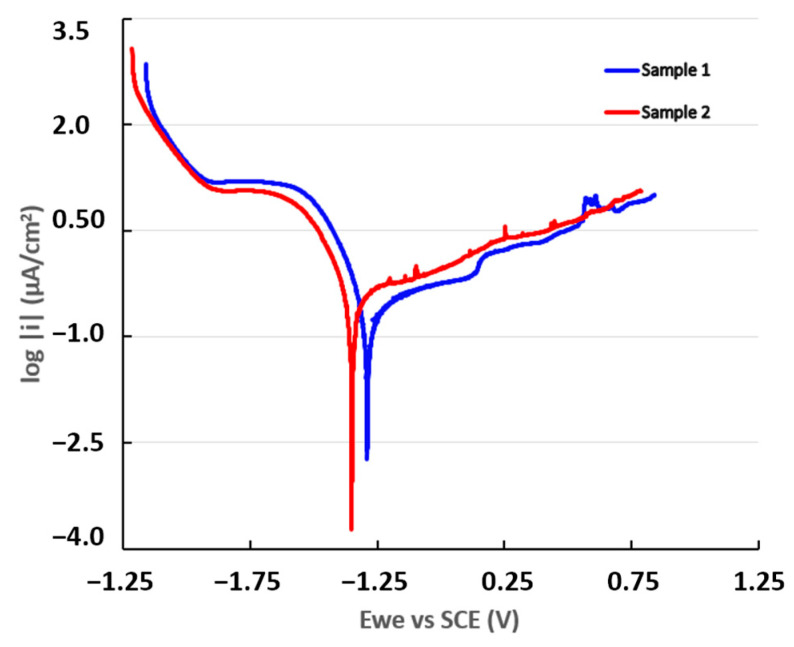
Potentiodynamic polarization curves for Sample 1 and Sample 2.

The potentiodynamic polarization curves were obtained during the tests carried out to estimate the corrosion rate (CR) of the samples. The current values are presented on a semi-logarithmic scale in Figure 5.

The potentiodynamic polarization curves showed an increase in anodic current densities with the addition of zirconium to Sample 2, indicating a decrease in corrosion resistance in the conditions of simulated seawater used in experimental tests. In the anodic range of both curves, small increases of the current were observed, suggesting the acceleration of the oxidation reaction due to local corrosion and repassivation processes.

The value of the cathodic current density decreased with the addition of zirconium. Consequently, this implies that, in the simulated environment used for testing, the zirconium acted as an inhibitor of the cathodic reaction, restricting the hydrogen evolution process.

The corrosion rate was calculated as follows:

(5) $$CR=\frac{I_{corr}KE_{W}}{\rho A},$$

where *I_corr_* is the corrosion current (A), *K* is the constant that defines the units of corrosion rate (3272 m/A·cm·year), *E_w_* is the equivalent weight (g/equivalent), *ρ* is the density (g/cm^3^), and *A* is the sample area (cm^2^).

Table 5 shows the electrochemical values obtained with these curves. The calculated CR data show values ranging from 2.44 × 10^−^^3^ mmpy for Sample 1 to 2.80 × 10^−^^3^ mmpy for Sample 2, representing a ~16-fold increase compared to Sample 1.

A more positive corrosion potential value can be observed for Sample 1 than for Sample 2. The corrosion current (i_corr_) is representative of the degree of oxidation of the alloy. A higher polarization resistance (R_p_) denotes that the alloy is more resistant to corrosion; thus, Sample 1 was more resistant to corrosion than Sample 2.

The Tafel slopes (βa and βc) were obtained through an analysis of the curve plotted in an interval of ±250 mV versus the open-circuit potential (OCP). Sample 1 showed a tendency toward passivation because it had a value of β_a_ greater than β_c_, while Sample 2 presented a corrosion tendency because the anodic slope was lower than the cathodic slope.

**Figure 6:**
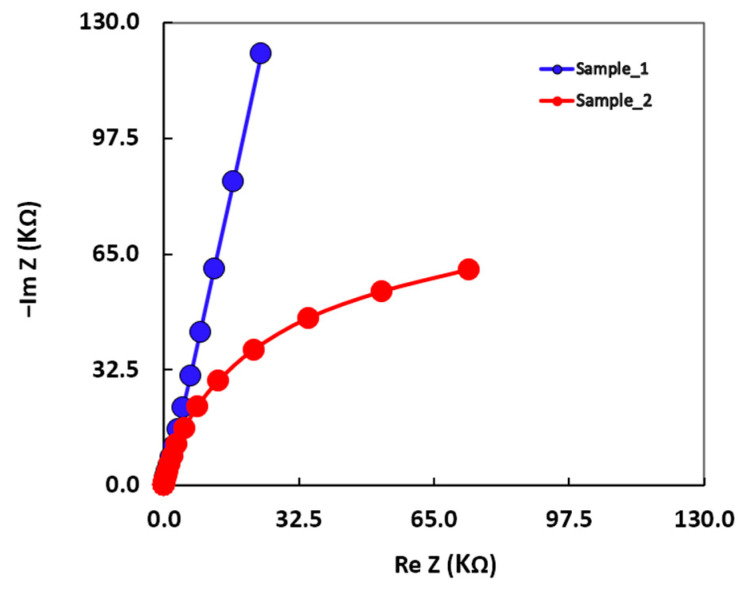
Nyquist diagrams for Sample 1 and Sample 2.

**Figure 7:**
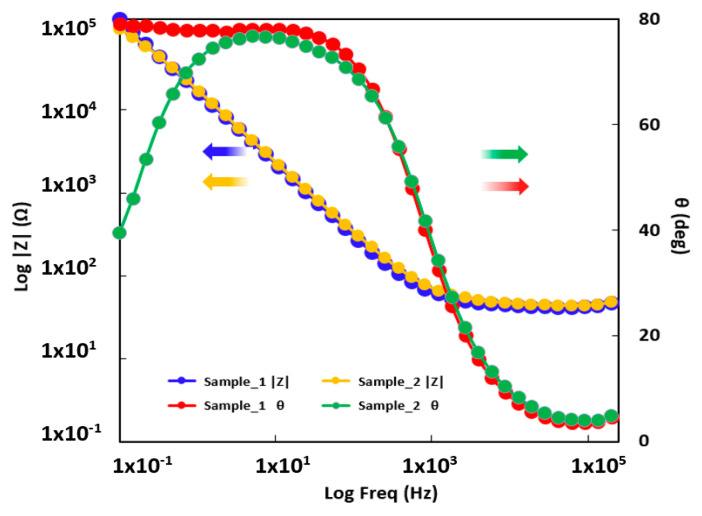
Bode diagrams for Sample 1 and Sample 2.

The characteristics of the oxide layer formed on an alloy surface can be estimated by means of the impedance technique, also known as electrochemical impedance spectroscopy (EIS). The graphs obtained from the EIS tests are presented as Nyquist plots in Figure 6 and Bode plots in Figure 7.

As can be observed in the Nyquist plots (see Figure 6), the radius of the semicircle for Sample 2 was smaller than that for Sample 1, indicating a low polarization resistance (a low corrosion resistance) for Sample 2.

In the Bode vs. |Z| plot (see Figure 7), a slight shift toward a higher value of the impedance module at the lowest frequency can be observed for Sample 1, indicating a slightly increased corrosion resistance of this alloy. In the Bode phase plots shown in Figure 6, a specific performance of the growth of a passive film can be observed for both alloys. This passive layer had a capacitive behavior with a phase angle approaching 90°. In the case of Sample 1, the higher phase angle was constant in a wide frequency band, a phenomenon related to an increase in the effective surface area.

After analyzing the shapes of the impedance diagrams, the experimental results could be fitted to an appropriate physical pattern consisting of an equivalent electrical circuit (EC). This circuit consists of several series or parallel configurations of resistors, capacitors, Warburg elements, etc. and provides the most relevant corrosion parameters of the substrate/electrolyte system. The equivalent circuit is similar to that proposed for Ti−xMo, Al_x_CoCrFeNi alloys, TiO_2_ nanofibers, and polymer electrolytes [45,46,47].

The equivalent circuit used to fit the experimental impedance data is presented in Figure 7, and the values of the corresponding elements are shown in Table 5.

**Figure 8:**
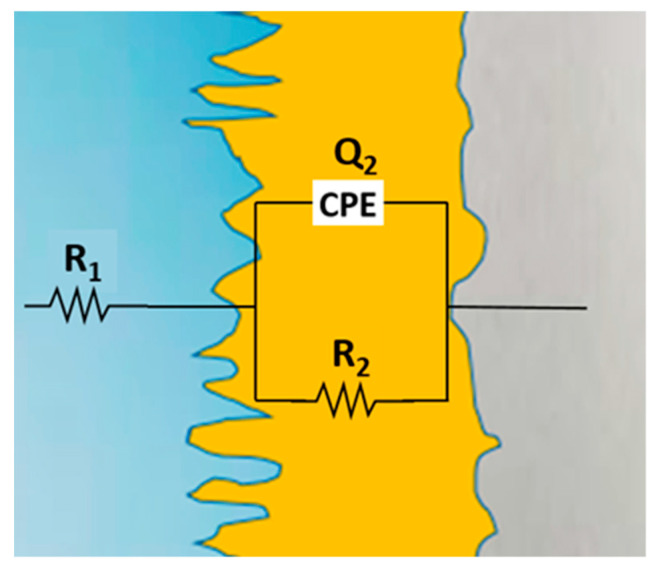
Equivalent circuit to fit the impedance data.

Within the circuit presented in Figure 8, the ohmic resistance of the simulated seawater is labeled R_1_, the resistance of the passive layer is labeled R_2_, and the capacitance of the passive layer is represented as Q_2_. As a consequence of the heterogeneous thin oxide film built up on the surface of the HEA alloys and the remarkable deviations of the Bode diagrams, it was required to replace the “ideal” capacitance by a constant-phase element (CPE) [48], the impedance of which is given [49] by Z = (*jω*)^−n^Y^0^, where *j* is an imaginary number (*j*^2^ = −1), *ω* is the angular frequency (rad·^s−1^), Y^0^ is the constant of the CPE [S(s·rad^−^^1^)^n^], n is the power number denoting the drift from ideal performance, n = α(π/2), and α is the constant-phase angle of the CPE (rad). Therefore, one of the parameters obtained when modeling the process is the ideality coefficient “*n*”, in such a way that the answer of the real process is more similar to the ideal as the value of n gets closer to unity and, consequently, the surface is more uniform. Thus, for n = 1, the CPE element is reduced to a capacitor with a capacitance Y^0^ and, if n = 0, to a simple resistor [50].

(6) $$\frac{1}{Z_{eq}}=\frac{1}{Z_{R_{2}}}+\frac{1}{Z_{Q_{2}}}.\;$$

With the aim of estimating the total impedance of the equivalent circuit, we computed the admittance of the parallel arrangement (*R_2_Q_2_*) as follows [51]:

Although a constant-phase element was used to fit the experimental results, the achieved value was considered as the capacity in the following equation:

(7) $$\frac{1}{Z_{eq}}=\frac{1}{R_{2}}+jwC_{2}.\;$$

Multiplying by *R_ct_*, we get

(8) $$Z_{eq}=\frac{R_{2}-j\left(wC_{2}R_{2}{}^{2}\right)}{1+{\left(wC_{2}R_{2}\right)}^{2}}.\;$$

Once the electrolyte ohmic resistance is added, the resulting impedance is

(9) $$Z_{eq}=R_{1}+\frac{R_{2}-j\left(wC_{2}R_{2}{}^{2}\right)}{1+{\left(wC_{2}R_{2}\right)}^{2}}.$$

*R*_2_ was taken as the corrosion resistance of the analyzed HEAs. The *R*_2_ values calculated by fitting the experimental data with the simulated results of the corresponding equivalent circuit are given in Table 6.

This decrease in *R*_2_ (and, consequently, in corrosion resistance) when adding Zr was due to the fact that the passive film formed on the surface of the HEAs changed its properties as a result of this addition and became thicker (Y^0^ decreased with Zr addition).

It can be noted that the passive film resistance *R*_2_ decreased with the addition of Zr because of the increase in the number of defective spots in the film. Without Zr, the passive film formed on the surface of the alloy was more compact and protective (see values of Y^0^ in the Table 6).

Doping with oversized atoms, such as zirconium, obstructs the grain coarsening of HEA, creating a supersaturated solid solution. Tekin et al. [38] observed the in situ formation of ZrO_2_ through TEM analysis; in contrast, in our XRD, although we had the peaks of the phases, these were not effective because the volume fraction of ZrO_2_ was below the detection limit of XRD. The formation of ZrO_2_ explained the high corrosion rate of Sample 2 with zirconium in comparison to Sample 1 without zirconium.

**Figure 9:**
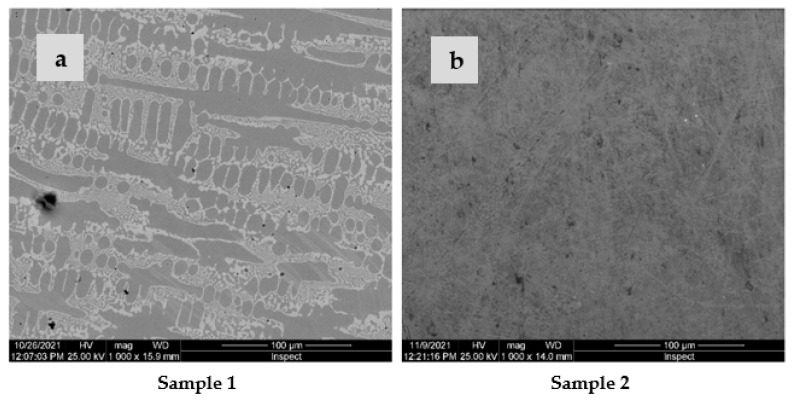
SEM image of the surface after performing the corrosion tests: (**a**) Sample 1 and (**b**) Sample 2.

After performing the corrosion tests, the surface was covered with a thin layer of oxide, which was subjected to SEM analyses, and the results are presented in Figure 9. The surface of both samples was covered with a very thin and transparent layer of oxide due to the chemical reactions during immersion in the corrosive solution. In the case of Sample 2 alloy, the oxide layer was thicker and further blurred the surface microstructure.

On the surface of Sample 1, a dendritic microstructure under the oxide layer and some corrosion pits were visible.

## 4. Conclusions

The effects of alloying with zirconium on the microstructure, elastic modulus, and corrosion properties of a high-entropy alloy, from the CoCrFeNiMo system, were investigated. A simulated corrosive solution (3.5% NaCl) was used to characterize the corrosion behavior of these materials using electrochemical techniques.

- A brittle fracture with a cleavage-like fracture plane could be observed for both alloys.
- The alloy without zirconium was more resistant to corrosion, with a very low corrosion rate (2.44 × 10^−3^ mmpy in comparison with 2.80 × 10^−3^ mmpy for the sample with Zr).
- The resistance of the passive film decreased with the addition of Zr (from 2.57 × 10^7^ kΩ·cm^2^ to 1.47 × 10^5^ kΩ·cm^2^) due to the increased number of defects points in the film.
- Doping with 0.71 wt.% Zr produced grain refinement and increased the hardness but did not increase the modulus of elasticity or corrosion resistance of the CoCrFeNiMo alloy.

## Figures and Tables

**Table 1:** Chemical composition of the experimental alloys.

Element	wt.%		at.%	
	Sample 1	Sample 2	Sample 1	Sample 2
Co	20.67	20.34	21.52	21.15
Cr	19.99	19.80	23.58	23.33
Fe	19.88	20.23	21.84	22.20
Mo	20.20	19.18	12.92	12.25
Ni	19.27	19.74	20.14	20.60
Zr	-	0.71	-	0.48

**Table 2:** Data of surface area, density and equivalent weight of specimens.

	S (cm^2^)	ρ (g/cm^3^)	*E_w_* (g/eq)
Sample 1	0.567 ± 0.002	8.635	23.694
Sample 2	0.676 ± 0.002	8.602	23.814

**Table 3:** EDS quantification results.

	Sample 1				Sample 2			
	Area 1		Area 2		Area 1		Area 2	
El.	wt.%	at.%	wt.%	at.%	wt.%	at.%	wt.%	at.%
Mo	13.86	8.85	30.55	20.61	13.64	8.51	26.07	17.13
Cr	19.23	22.07	20.42	25.12	19.28	22.2	20.58	24.96
Fe	22.02	23.53	16.29	18.66	22.21	23.81	17.97	20.29
Co	22.49	22.78	18.34	19.91	21.74	22.09	18.92	20.25
Ni	22.41	22.78	14.40	15.69	22.54	22.99	15.55	16.82
Zr	-	-	-	-	0.59	0.39	0.80	0.55

**Table 4:** Modulus of elasticity and hardness values for Sample 1 and Sample 2.

Alloy	Modulus of Elasticity (GPa)	Microhardness (HV0.2)
Sample 1	173.99 ± 4.25	369.2 ± 0.6
Sample 2	144.33 ± 5.38	402.6 ± 0.7

**Table 5:** Electrochemical parameters obtained from Tafel curves.

	E_corr_	i_corr_	β_c_	β_a_	R_p_	CR
	[mV]	[µA/cm^2^]	[mV/dec]	[mV/dec]	[kΩ·cm^2^]	[mmpy]
Sample 1	−288 ± 1	0.142 ± 0.012	104 ± 3	335 ± 5	150 ± 16	2.44 × 10^−3^
Sample 2	−346 ± 3	0.210 ± 0.032	121 ± 4	338 ± 2	113 ± 12	2.80 × 10^−3^

**Table 6:** Fitted EIS parameters using the equivalent circuit model presented in Figure 8.

	Sample 1	Error %	Sample 2	Error %
*R*_1_ (kΩ·cm^2^)	42.70	0.96	45.09	1.22
Q, Y^0^ (S·S^n^/cm^2^)	1.20 × 10^−5^	1.23	1.12 × 10^−5^	1.72
Q, n	0.88	0.28	0.87	0.38
*R*_2_ (kΩ·cm^2^)	2.57 × 10^7^	5.40	1.47 × 10^5^	4.75
χ^2^	1.18 × 10^−3^		1.87 × 10^−3^	

## Data Availability

For the data supporting reported results can be asked the authors.
